# Supplementary material for: COZOID: contact zone identifier for visual analysis of protein-protein interactions
Source: BMC Bioinformatics. 2018 Apr 6;19:125. doi: 10.1186/s12859-018-2113-6 (PMC5889581; doi:10.1186/s12859-018-2113-6)
Supplement: Supplementary file 2 — Software build. Executable binary file of the software tool. (ZIP 118,784 kb) [file 12859_2018_2113_MOESM2_ESM.zip › cozoid/platform/docs/testng-6.5.1-javadoc.zip!/org/testng/ITestMethodFinder.html]

ITestMethodFinder (TestNG)


---


|  |  |  |
| --- | --- | --- |
| **Overview** \| **Package** \| **Class** \| **Tree** \| **Deprecated** \| **Index** \| **Help** | |  |
| **PREV CLASS**   **NEXT CLASS** | **FRAMES**    **NO FRAMES**     **All Classes** |  |
| SUMMARY: NESTED \| FIELD \| CONSTR \| METHOD | DETAIL: FIELD \| CONSTR \| METHOD |  |


---


## org.testng Interface ITestMethodFinder

---

``` public interface ITestMethodFinder ```

This interface allows to modify the strategy used by TestRunner
to find its test methods. At the time of this writing, TestNG
supports two different strategies: TestNG (using annotations to
locate these methods) and JUnit (setUp()/tearDown() and all
methods that start with "test" or have a suite() method).

**Author:**
:   Cedric Beust, May 3, 2004

---

| **Method Summary** | |
| --- | --- |
| `ITestNGMethod[]` | `getAfterClassMethods(java.lang.Class cls)` |
| `ITestNGMethod[]` | `getAfterGroupsConfigurationMethods(java.lang.Class testClass)` |
| `ITestNGMethod[]` | `getAfterSuiteMethods(java.lang.Class cls)` |
| `ITestNGMethod[]` | `getAfterTestConfigurationMethods(java.lang.Class testClass)` |
| `ITestNGMethod[]` | `getAfterTestMethods(java.lang.Class cls)` |
| `ITestNGMethod[]` | `getBeforeClassMethods(java.lang.Class cls)` |
| `ITestNGMethod[]` | `getBeforeGroupsConfigurationMethods(java.lang.Class testClass)` |
| `ITestNGMethod[]` | `getBeforeSuiteMethods(java.lang.Class cls)` |
| `ITestNGMethod[]` | `getBeforeTestConfigurationMethods(java.lang.Class testClass)` |
| `ITestNGMethod[]` | `getBeforeTestMethods(java.lang.Class cls)` |
| `ITestNGMethod[]` | `getTestMethods(java.lang.Class cls, XmlTest xmlTest)` |

**Method Detail**

### getTestMethods

```
ITestNGMethod[] getTestMethods(java.lang.Class cls,
                               XmlTest xmlTest)
```

:   **Returns:**: All the applicable test methods.

---


### getBeforeTestMethods

```
ITestNGMethod[] getBeforeTestMethods(java.lang.Class cls)
```

:   **Returns:**: All the methods that should be invoked before a test method is invoked.

---


### getAfterTestMethods

```
ITestNGMethod[] getAfterTestMethods(java.lang.Class cls)
```

:   **Returns:**: All the methods that should be invoked after a test method completes.

---


### getBeforeClassMethods

```
ITestNGMethod[] getBeforeClassMethods(java.lang.Class cls)
```

:   **Returns:**: All the methods that should be invoked after the test class has been created and before any of its test methods is invoked.

---


### getAfterClassMethods

```
ITestNGMethod[] getAfterClassMethods(java.lang.Class cls)
```

:   **Returns:**: All the methods that should be invoked after the test class has been created and after all its test methods have completed.

---


### getBeforeSuiteMethods

```
ITestNGMethod[] getBeforeSuiteMethods(java.lang.Class cls)
```

:   **Returns:**: All the methods that should be invoked before the suite starts running.

---


### getAfterSuiteMethods

```
ITestNGMethod[] getAfterSuiteMethods(java.lang.Class cls)
```

:   **Returns:**: All the methods that should be invoked after the suite has run all its tests.

---


### getBeforeTestConfigurationMethods

```
ITestNGMethod[] getBeforeTestConfigurationMethods(java.lang.Class testClass)
```

---


### getAfterTestConfigurationMethods

```
ITestNGMethod[] getAfterTestConfigurationMethods(java.lang.Class testClass)
```

---


### getBeforeGroupsConfigurationMethods

```
ITestNGMethod[] getBeforeGroupsConfigurationMethods(java.lang.Class testClass)
```

---


### getAfterGroupsConfigurationMethods

```
ITestNGMethod[] getAfterGroupsConfigurationMethods(java.lang.Class testClass)
```


---


|  |  |  |
| --- | --- | --- |
| **Overview** \| **Package** \| **Class** \| **Tree** \| **Deprecated** \| **Index** \| **Help** | |  |
| **PREV CLASS**   **NEXT CLASS** | **FRAMES**    **NO FRAMES**     **All Classes** |  |
| SUMMARY: NESTED \| FIELD \| CONSTR \| METHOD | DETAIL: FIELD \| CONSTR \| METHOD |  |


---
